# Pharmacokinetics, Safety, and Tolerability of Tenapanor in Healthy Chinese and Caucasian Volunteers: A Randomized, Open-Label, Single-Center, Placebo-Controlled Phase 1 Study

**DOI:** 10.1155/2024/1386980

**Published:** 2024-03-06

**Authors:** Gang Yuan, Yili Chen, Li Li, Xin Wang, Gang Wei, Jiawei Zeng, Ai-Min Hui, Yueyun Jiang, Han Zhao, Lei Diao, Yongchun Zhou, Yinglian Xiao, Minhu Chen

**Affiliations:** ^1^The First Affiliated Hospital, Sun Yat-sen University, Guangzhou, Guangdong, China; ^2^Global R&D Center, Shanghai Fosun Pharmaceutical Development, Co., Ltd, Shanghai, China; ^3^Wanbang Biopharmaceuticals, Xuzhou, Jiangsu, China

## Abstract

**Background:**

Tenapanor is a locally acting selective sodium-hydrogen exchanger 3 inhibitor with the potential to treat sodium/phosphorus and fluid overload in various cardiac-renal diseases, which has been approved for constipation-predominant irritable bowel syndrome in the US. The pharmacokinetics (PK) of tenapanor and its metabolite tenapanor-M1 (AZ13792925), as well as the safety and tolerability of tenapanor, were investigated in healthy Chinese and Caucasian subjects.

**Methods:**

This randomized, open-label, single-center, placebo-controlled phase 1 study (https://www.chinadrugtrials.org.cn; CTR20201783) enrolled Chinese and Caucasian healthy volunteers into 4 parallel cohorts (3 cohorts for Chinese subjects, 1 cohort for Caucasian subjects). In each cohort, 15 subjects were expected to be included and received oral tenapanor (10 or 30 mg as single dose, or 50 mg as a single dose followed by a twice-daily repeated dose from Day 5 to 11, with a single dose in the morning on Day 11) or placebo in a 4 : 1 ratio.

**Results:**

59 healthy volunteers received tenapanor 10 mg (*n* = 12 Chinese), 30 mg (*n* = 12 Chinese), or 50 mg (*n* = 12 (Chinese), *n* = 11 (Caucasian)) or placebo (*n* = 12, 3 per cohort). After single and twice-daily repeated doses, tenapanor plasma concentrations were all below the limit of quantitation; tenapanor-M1 appeared slowly in plasma. In single-ascending dose evaluation (10 to 50 mg) of Chinese subjects, the mean *C*_max_, AUC_0-*t*_, and AUC_0-∞_ of tenapanor-M1 increased with increasing dose level, and AUC_0-*t*_ increased approximately dose proportionally. The *C*_max_ accumulation ratio was 1.55 to 6.92 after 50 mg repeated dose in Chinese and Caucasian subjects. Exposure to tenapanor-M1 was generally similar between the Chinese and Caucasian subjects. Tenapanor was generally well-tolerated and the safety profile was similar between the Chinese and Caucasian participants receiving tenapanor 50 mg, as measured by vital signs, physical and laboratory examination, 12-lead ECG, and adverse events. No serious adverse event or adverse event leading to withdrawal occurred.

**Conclusion:**

Tenapanor was well-tolerated, with similar PK and safety profiles between Chinese and Caucasian subjects. This trial is registered with CTR20201783.

## 1. Background

Globally, the prevalence of all-stage chronic kidney disease (CKD) was 9.1% in 2017. A substantial increase was noted in the incidence of end-stage renal disease (ESRD) in individuals receiving renal replacement therapy [1]. The ability to excrete phosphorus gradually diminishes with the progressive impairment of renal function. Consequently, hyperphosphatemia is encountered commonly in ESRD patients.

Hyperphosphatemia is known to be associated with vascular calcification, cardiovascular events, and mortality among patients with CKD, especially in those receiving maintenance hemodialysis [2–4]. Therefore, practice guidelines recommend that serum phosphate levels should be maintained within the normal range, i.e., 3.5 to 5.5 mg/dL [5, 6]. However, due to the complexity of phosphate metabolism regulation and the practical challenges of currently available therapeutic modalities, the management of hyperphosphatemia remains unsuccessful in many patients [7, 8]. The development of oral phosphorus-lowering drugs that possess novel mechanisms of action which allow for easier delivery and improved benefit-risk profiles is needed urgently.

Irritable bowel syndrome (IBS) is a common functional gastrointestinal disorder that is associated with a significant healthcare burden; for some patients, IBS symptoms can be disabling [9]. The prevalence of IBS ranges from 2% to 6% in most countries [10]. IBS patients are categorized as those having a tendency for predominant diarrhea (IBS-D) or those with predominant constipation (IBS-C). Features of IBS pathogenesis include changes in fecal microbial flora and gastrointestinal motility, post-infectious reactivity, brain-gut interactions, overgrowth of bacteria, sensitivity to food, malabsorption of carbohydrate, and intestinal inflammation [11]. Increased understanding of the molecular and pathophysiological pathways of IBS has led to the development of new pharmacological agents and the identification of potential novel therapeutic targets [9].

The sodium-hydrogen exchanger 3 (NHE3) is important for intestinal sodium transport and subsequent fluid homeostasis. NHE3 inhibition in the gut by tenapanor, a minimally absorbed selective NHE3 inhibitor, decreases gastrointestinal sodium absorption which results in an elevation in stool fluid content [12] and a reduction in the paracellular absorption of luminal phosphate [13, 14]. Thus, tenapanor may be useful in the treatment of sodium/phosphorus and fluid overload in various cardiac-renal diseases as well as in constipation-related disorders.

Following on from positive results obtained in the phase 3 T3MPO trial, tenapanor was approved by the US Food and Drug Administration for the treatment of adults with IBS-C [15]. In phase 1 to phase 3 trials, tenapanor, both as monotherapy and in combination with an established phosphate binder regimen, significantly reduced serum phosphorus concentrations in hyperphosphatemic patients who were receiving maintenance dialysis [16–19]. Tenapanor monotherapy reduced concentrations of serum phosphorus by a similar magnitude to that of an established phosphate binder [17, 19].

Potential differences in pharmacokinetics across ethnic groups may affect drug efficacy and safety and the feasibility of generalizing the findings of pivotal studies to particular populations. To date, the pharmacokinetics (PK) and safety profile of tenapanor in the Chinese population have not been established. Here, we report the PK of tenapanor and its major inactive metabolite, tenapanor-M1 (AZ13792925), as well as the tolerability and safety of tenapanor, in healthy Chinese and Caucasian individuals in a phase 1 study.

## 2. Materials and Methods

### 2.1. Study Design

This was an open-label, single-center, randomized, placebo-controlled phase 1 study (Identifier: CTR20201783). In this study, 60 Chinese and Caucasian healthy volunteers were expected to be recruited, who were enrolled into 4 parallel cohorts (cohort 1 to 3 for Chinese subjects, cohort 4 for Caucasian subjects, *n* = 15 in each cohort). For cohorts 1 and 2, participants were randomized in a 4 : 1 ratio to receive one single dose of tenapanor (10 or 30 mg) or placebo. For cohorts 3 and 4, participants received tenapanor 50 mg (or the corresponding placebo, in a 4 : 1 ratio) as a single dose on Day 1, followed by a repeated dose of the same tablets (twice daily, from Day 5 to 11, with only a single dose given in the morning on Day 11). The study was conducted according to the principles contained in the Declaration of Helsinki. All participants provided the written informed consent.

### 2.2. Participants

Chinese or Caucasian volunteers were eligible if they were (1) a resident of China; (2) able to understand and adhere to the protocol, with written informed consent; (3) aged between 18 and 45 years; (4) not less than 45 kg (female) or 50 kg (male) in body weight with a body mass index (BMI) of 18.5 to 28.0 kg/m^2^; (5) healthy, as measured by vital signs, physical and laboratory examination, 12-lead electrocardiography (ECG), chest X-ray, and infectious disease screening in the 2-week period before inclusion. A negative pregnancy test was required for female subjects.

Key exclusion criteria included the following: a history or presence of clinically significant hepatic, gastrointestinal, or renal disease, including gastrointestinal surgery (other than appendectomy) or any other condition known to interfere with the study drugs; loose stools (Bristol Stool Form Scale score [20] of 6 or 7) for ≥2 days in the 7 days before inclusion; use of medications or supplements in the 14-day period before assignment to treatment; and abuse of tobacco, alcohol, or medications.

### 2.3. Study Drug Administration

Subjects were screened within 14 days prior to the baseline period. After enrollment, subjects were admitted to the Phase I Clinical Trial Center at the First Affiliated Hospital, Sun Yat-sen University, Guangzhou, Guangdong, China, and were given a randomization number.

In cohort 1 (10 mg) and cohort 2 (30 mg), Chinese subjects received either tenapanor (one or three tablets of tenapanor 10 mg, respectively) or placebo on Day 1. In cohort 3 (50 mg-Chinese) and cohort 4 (50 mg-Caucasian), subjects received one tablet of tenapanor 50 mg or placebo on Day 1, followed by a repeated dose with the same tablet twice daily for 7 days (from Day 5 to 11, with only a single dose given in the morning on Day 11).

Tenapanor tablets or placebo were delivered with 240 mL of water in the morning of the next day (Day 1) after at least 10 hours of fasting. For all single-dose evaluations, tablets were administrated 5 to 10 minutes before breakfast on Day 1. For the repeated-dose evaluation, tablets were administrated 5 to 10 minutes before breakfast and supper from Day 5 to Day 11 (only in the morning on Day 11). Standard meals were provided by the clinical center. Subjects took the assigned food at the appointed time during the hospital stay and did not take any other diet.

### 2.4. Study Endpoint and Assessments

The primary endpoint was the PK parameters of tenapanor and its major inactivate metabolite, tenapanor-M1, in Chinese and Caucasian subjects. For single-dose evaluation, PK profile parameters included peak plasma concentration (*C*_max_), time to reach *C*_max_ (*T*_max_), area under the concentration-time curve from time zero to the last measurable concentration (AUC_0-*t*_) and total AUC (AUC_0-∞_), elimination half-life (*t*_1/2_), apparent clearance (CL/F), and apparent volume of distribution (*V*_*d*_/*F*). For repeated-dose evaluation, parameters included *T*_max_ (*T*_max ,ss_), plasma concentration (*C*_ss_), *C*_max_ (*C*_max ,ss_), average plasma concentration (*C*_av,ss_), trough concentration (*C*_min ,ss_), and CL/F (CL_ss_/F) at steady state, as well as AUC at steady state over the dosing interval (AUC_0-*τ*_), *t*_1/2_, and degree of fluctuation (DF). Secondary endpoints included safety and tolerability, as measured by vital signs, physical and laboratory examination, 12-lead ECG, and adverse events (AEs).

Blood samples for PK analysis were collected within 1 hour at pre-dose and at 1, 2, 4, 6, 7, 8, 9, 10, 12, 24, 48, and 72 hours post-dose, on Day 1 for single-dose analysis, or on Day 11 for repeated-dose analysis. Plasma concentrations of tenapanor and tenapanor-M1 were quantified by liquid chromatography-tandem mass spectrometry (LC-MS/MS; Sciex Triple Quad 6500+, Framingham, MA, USA) using a HPLC column (Poroshell HPH-C18, 4 *µ*m, 4.6 × 50 mm, Agilent, Santa Clara, CA, USA), and mobile phase A (5 mM ammonium formate and 0.1% formic acid in purified water) and B (0.1% formic acid in acetonitrile), maintained at 40°C. The gradient elution was delivered as follows (A : B): 0.01–3.00 min: 70 : 30–40 : 60; 3.00–3.05 min: 40 : 60–5 : 95; 3.05–4.50 min: 5 : 95–5 : 95; 4.50–4.55 min: 5 : 95–70 : 30. The flow rate was 0.6 mL/min for 0–3.05 min, 1.2 mL/min for 3.10–4.50 min, and 0.6 mL/min for 4.55–6.00 min. Isotope-labeled tenapanor-d8 and [13C]6-M1 were used as internal standards. The lower and upper limits of quantification were 0.5 and 300 ng/mL for tenapanor and M1, respectively. AEs were recorded according to the Medical Dictionary for Regulatory Activities (MedDRA) version 24.1. Vital signs including blood pressure, heart rate, and body temperature were collected at screening, during the study and before subject discharge. Physical and laboratory examinations, as well as ECGs, were conducted at screening and before subject discharge.

### 2.5. Statistical Analysis

PK analysis was conducted according to the noncompartmental method using Phoenix software (version 8.3, Pharsight Corporation, Mountain View, CA, USA). SAS (version 9.4, SAS Institute Inc., Cary, NC, USA) was used for statistical analysis.

For each group, continuous data were summarized using the number of subjects, mean, standard deviation (SD), median, and minimum and maximum values. Categorical data were summarized using frequencies and percentages. Geometric mean and coefficient of variation (CV) were used for applicable plasma concentrations and PK parameters. The linear PK characteristics of PK parameters were evaluated by a power function model based on confidence interval. Dose proportionality for *C*_max_, AUC_0-*t*_, and AUC_0-∞_ was evaluated by power function model, according to Hummel standard (judgment interval: [1 + ln(*θ*_*L*_)/ln(*r*), 1 + ln(*θ*_*H*_)/ln(*r*)], *θ*_*L*_ = 0.5, *θ*_*H*_ = 2.0 and *r* = maximum dose/minimum dose) based on comparing the 95% confidence interval for the ratio of predicted mean values from the extremes of the dose range to pre-defined equivalence criterion [21–23].

Due to the large variation in PK parameters of tenapanor and the small number of subjects in each group, statistical analysis of PK parameters was mainly descriptive with no formal statistical assumption. A comparison of ethnic differences in the PK profile was conducted in Chinese and Caucasian subjects in the 50 mg dose group, based on box-and-whisker plots of the main PK parameters, the geometric mean ratio (GMR) of parameters between different ethnic groups, and their 90% confidence intervals (PK parameters were considered similar in the two ethnic groups if the 90% confidence intervals for the GMRs of PK parameters crossed 1.00 as well as the point estimate of the GMRs were between 0.80 and 1.25), combined with clinical relevance.

## 3. Results

### 3.1. Study Participants

Of 310 healthy subjects screened, 59 subjects were enrolled in the study, including 45 Chinese and 14 Caucasian subjects. The mean (±SD) age of subjects was 27.4 ± 5.04 years, 39 (66.1%) were male. In general, the demographic and baseline characteristics of subjects were well balanced across all treatment groups (Table 1).

### 3.2. Pharmacokinetics

#### 3.2.1. Single-Ascending Dose (SAD)

**Figure 1:**
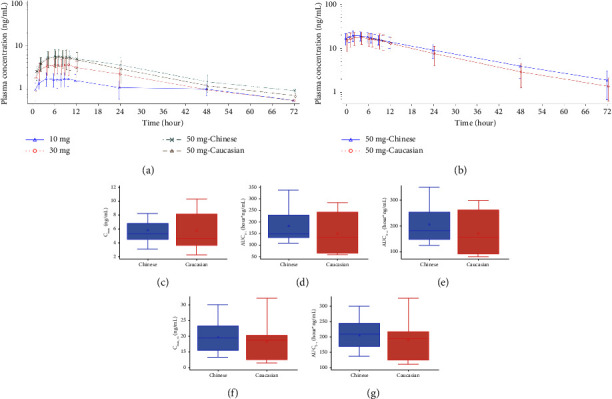
Pharmacokinetic characteristics of tenapanor-M1 following administration of tenapanor. (a, b) Mean plasma concentration of tenapanor-M1 (semi-log plot) following single dose (a) and repeated dose (b). (c–g) *C*_max_ and AUC of tenapanor-M1 following 50 mg single dose (c–e) and repeated dose (f, g) administration of tenapanor in Chinese and Caucasian subjects. The five horizontal lines from the bottom to the top of the box with whisker indicate minimum without outliers, *Q*1, median, *Q*3, and maximum without outliers. The triangle or circle inside the box represents the mean value. The triangle or circle outside the box represents the potential outliers.

The tenapanor plasma concentration was below the lower limit of quantitation (LLOQ, 0.5 ng/mL) in all Chinese and Caucasian subjects receiving a single dose of tenapanor 10, 30, or 50 mg. The major inactive metabolite, tenapanor-M1, appeared slowly in plasma following SAD administration of tenapanor 10 to 50 mg, with a median *T*_max_ between 7 and 8.458 hours (Figure 1(a); Table 2). The plasma concentration of tenapanor-M1 increased with increasing dose levels. In SAD evaluation (10 mg to 50 mg in Chinese subjects), the slope of AUC_0-*t*_ was 0.98, close to 1, and the 95% confidence interval was 0.74 to 1.22. It falls within the target interval of 0.57 to 1.43, which indicated that AUC_0-*t*_ of tenapanor-M1 from 10 mg to 50 mg was approximately linear, according to Hummel standard [21–23]. While AUC_0-∞_ and *C*_max_ increased slightly less than dose proportionally (Table 3 and Supplementary Figure 1). After the administration of tenapanor, tenapanor-M1 had a long half-life, with a geometric mean *t*_1/2_ between 16.37 and 20.61 hours. After a single dose of tenapanor 10 mg, 30 mg, or 50 mg in Chinese subjects, the geometric mean *C*_max_ of tenapanor-M1 was 1.7294, 3.4924, and 5.5194 ng/mL, respectively, and the geometric mean AUC_0-*t*_ was 34.1165, 90.9420, and 169.6988 hour *∗* ng/mL, respectively. The geometric mean *C*_max_ and AUC_0-*t*_ of tenapanor-M1 were 5.2455 ng/mL and 129.4200 hour *∗* ng/mL, respectively, after a single dose of tenapanor 50 mg in Caucasian subjects.

#### 3.2.2. Repeated Dose

The plasma concentration of tenapanor was below the LLOQ in all Chinese and Caucasian subjects receiving tenapanor 50 mg twice daily. Based on the apparent terminal elimination half-life of tenapanor-M1, it was suggested that steady state reached within 4 to 5 days after repeated dosing. The median *T*_max_ of tenapanor-M1 was 1.975 (range: 0.98 to 6.97) and 4 (range: 0.00 to 9.00) hours at steady state in Chinese and Caucasian subjects, respectively. Following repeated doses of tenapanor 50 mg twice daily, the geometric mean *C*_max −ss_ and AUC_0-*τ*_ of tenapanor-M1 were 19.1772 ng/mL and 201.7129 hour *∗* ng/mL in Chinese subjects, respectively, and 17.6014 ng/mL and 182.5681 hour *∗* ng/mL in Caucasian subjects (Figure 1(b); Table 2), respectively. The geometric mean accumulation ratio of *C*_max_ was 3.47 (range, 1.55 to 6.78) in Chinese subjects and 3.36 (range, 1.98 to 6.92) in Caucasian subjects, after repeated doses of tenapanor 50 mg.

#### 3.2.3. Ethnic Differences

Exposure to tenapanor-M1, as assessed by mean *C*_max_ and mean AUC, was generally similar in Chinese and Caucasian subjects after administration of tenapanor 50 mg as single or repeated doses (Figure 1). The geometric mean ratio (GMR) of *C*_max_ in Chinese subjects relative to Caucasian subjects was 1.05 (90% CI, 0.78 to 1.41) after tenapanor 50 mg as a single dose and 1.09 (90% CI, 0.88 to 1.34) after repeat-dose tenapanor 50 mg. The GMR of AUC_0-*t*_ was 1.31 (90% CI, 0.92 to 1.87) and the GMR of AUC_0-∞_ was 1.13 (90% CI, 0.84 to 1.53) after single-dose tenapanor 50 mg. The GMR of AUC_0-*τ*_ was 1.10 (90% CI, 0.89 to 1.37) after repeat-dose tenapanor 50 mg. PK parameters in Chinese and Caucasian subjects after single and repeated doses of tenapanor 50 mg are shown in Supplementary Table 1.

### 3.3. Safety and Tolerability

No significant changes or trends in laboratory tests, vital signs, ECG, or physical examination findings were observed after single and/or repeated doses of tenopanor in each dose cohort.

AEs that occurred in each treatment group are summarized in Table 4. The incidence of treatment-emergent AEs (TEAEs) of any grade was 41.7%, 58.3%, and 100% in subjects receiving tenapanor 10, 30, or 50 mg (single or repeated doses). Six of 12 (50%) subjects in the placebo group experienced at least 1 TEAE.

TEAEs reported most commonly in subjects receiving tenapanor at any dose level were gastrointestinal disorders, including diarrhea, abdominal distention, upper abdominal pain, and dyspepsia. Diarrhea was considered a tenapanor treatment-related AE (TRAE) and occurred more frequently in subjects receiving tenapanor 50 mg compared to those who received placebo. The incidence of diarrhea and other gastrointestinal disorders was similar in Chinese and Caucasian subjects receiving tenapanor 50 mg. The incidences of other AEs of interest, such as elevated systolic blood pressure, elevated transaminases, hyponatremia, and vertigo, were similar in the tenapanor and placebo groups. All TEAEs were mild in severity and were resolved by the end of the follow-up period. No serious AEs (SAEs) or AEs leading to withdrawal occurred during the study.

## 4. Discussion

An important feature of the gastrointestinal tract is to maintain a stable internal environment through balancing the secretion and absorption of fluids and sodium. While the role of NHE3 in intestinal sodium absorption is critical, the mechanism of action of tenapanor to target NHE3 in the gastrointestinal tract results in an increase in stool fluid content as well as a reduction in the absorption of luminal phosphate [12]. This important feature of tenapanor forms the basis for treating various diseases, including constipation-related disorders and hyperphosphatemia.

In our study, we characterized the PK of tenapanor and its major inactivate metabolite in Chinese and Caucasian healthy volunteers. The tenapanor plasma concentration was below the LLOQ in all blood samples collected from all subjects up to a dose of 50 mg, aligning with data reported from other studies in healthy volunteers [12, 16, 24]. In contrast, the major inactive metabolite, tenapanor-M1, was detectable and appeared slowly in plasma. In SAD evaluation, the AUC_0-*t*_ of tenapanor-M1 from 10 mg to 50 mg was approximately linear [21–23], while AUC_0-∞_ and *C*_max_ increased in a manner that was slightly less than dose proportional. The PK profile of tenapanor-M1 was numerically similar with the results observed in a phase 1 Japanese study, in which the median *T*_max_ was about 4 to 8 hours after single or repeated tenapanor doses of 15 to 90 mg, with an accumulation ratio of *C*_max_ of 5.00 to 8.18 after repeated doses (at multiple dose levels), and the average *C*_max_ and AUC of tenapanor-M1 also increased in an approximately dose proportional way (unpublished data; Supplementary Figure 2). Moreover, the *C*_max_ in healthy Japanese subjects was close to that in healthy Chinese subjects, with geometric (CV%) *C*_max_ values of 2.47 (53.3) and 3.49 (38.18) ng/mL, respectively, after tenapanor 30 mg as a single dose.

Overall, the PK profiles of tenapanor in Chinese and Caucasian subjects were similar. The 90% CI of the *C*_max_ and AUC GMRs all crossed 1.00. Most of the GMR point estimates of the PK exposure parameters (i.e., *C*_max_ and AUC_0-∞_ after single oral dose and *C*_ss_, *C*_max,ss_, *C*_min,ss_, *C*_av,ss_, and AUC_0-*τ*_ after repeated oral dose) for Chinese or Caucasian subjects were within the range of 0.80 to 1.25. The only exception was the point estimate of the GMR for AUC_0-*t*_ of tenapanor-M1 after single oral administration, which was 1.31; this may be partially explained by the small sample size, the uneven number of cases, and the low tenapanor-M1 plasma concentration, which was already lower than the LLOQ in some subjects at 48 or 72 hours. Moreover, minor differences in tenapanor-M1 exposure levels between Chinese and Caucasian subjects did not cause clinically meaningful differences in safety between the two groups. Our observations were in line with data reported from other studies, such as in a phase 1 study showing that the safety profile and pharmacodynamic effects of tenapanor were similar in Caucasian and Japanese healthy volunteers [16]. In addition, exposure to tenapanor-M1 (as assessed by mean *C*_max_ and AUC_0–8_) for Japanese and Caucasian subjects was generally similar at the 90 mg twice daily dose (unpublished data).

In our study, tenapanor showed a good safety profile in healthy volunteers. At 10, 30, and 50 mg single-dose and 50 mg repeated-dose levels, tenapanor was generally well-tolerated in Chinese and Caucasian healthy volunteers. The most commonly reported AEs were gastrointestinal disorders; diarrhea was the most common event and this is consistent with the findings from other studies in healthy volunteers [12, 16, 24]. According to the reported study in Japanese population [16], two events of treatment-related diarrhea (60 and 90 mg twice daily, one per group, 8.3%) occurred in healthy Japanese subjects. While 12/12 (100%) events of all-cause diarrhea occurred in 50 mg twice daily group in Chinses subjects in our study. This discrepancy is probably due to the different criteria for evaluating diarrhea as well as the fact that the Japanese study also examined the pharmacodynamics of tenapanor, where the subjects were more restricted to standard meals, which may affect the incidence of diarrhea. As a result of the pharmacological effect of tenapanor, leading to increased gastrointestinal tract water retention, diarrhea is an expected AE. All reported diarrhea events in this study were mild in severity and resolved with symptomatic intervention (such as montmorillonite), which is also consistent with the observation of rare discontinuations or dose adjustment of tenapanor due to diarrhea in other studies [12, 16, 17, 25].

The safety of tenapanor in patients with renal impairment has been evaluated in three phase 3 studies including patients with ESRD who were receiving maintenance hemodialysis [17, 18]. In the TEN-02-201 trial, TRAEs were reported in 46.5% of patients receiving tenapanor 30 mg twice daily in the randomized treatment period. SAEs occurred in 7.0% of patients, and 15.5% of patients experienced AEs leading to discontinuation [17]. In the AMPLIFY trial, treatment-related diarrhea occurred in 40.2% of patients receiving tenapanor + phosphate binder, compared with 6.7% of patients in the placebo + phosphate binder group. AEs leading to study drug discontinuation occurred in 4.3% and 2.5% of patients in the two groups, respectively [18]. The safety of tenapanor in the Chinese population with renal impairment was similar with the studies of TEN-02-201 and AMPLIFY. Diarrhoea was the predominant TRAE (28.6%) and most was mild in severity [26].

CKD has become an important public health burden in China [27]. Given that hyperphosphatemia is an important complication in CKD patients that can result in unfavorable clinical consequences, there is currently an unmet need for effective and safe phosphorus-lowering drugs which act through novel mechanisms, especially for the vast number of Chinese patients. As demonstrated in pivotal phase 2 and 3 clinical trials, tenapanor, both as monotherapy and in combination with an established phosphate binder regimen, decreased concentrations of serum phosphorus significantly in patients who were receiving maintenance dialysis [17–19, 25, 26].

There were some limitations in our study. As a molecule that acts locally in the intestine, tenapanor absorption into the circulation is known to be minimal. Tenapanor-M1, the only major inactive metabolite of the prototype drug tenapanor, was quantifiable in plasma at a low concentration. Thus, the PK comparison data should be interpreted with caution. Nevertheless, our results and conclusions are in accordance with previous observations from a study which compared Japanese and Caucasian subjects [16]. Second, the PK and safety profile of a repeated dose of tenapanor were not evaluated at lower dose levels (e.g., 30 mg).

## 5. Conclusions

Tenapanor was safe and well-tolerated in healthy Chinese volunteers. Similar PK and safety profiles were observed between Chinese and Caucasian subjects. These results support further clinical investigations of tenapanor in the Chinese population.

## Figures and Tables

**Table 1:** Demographic and baseline characteristics of Chinese and Caucasian participants.

Characteristics	Tenapanor 10 mg (*n* = 12)	Tenapanor 30 mg (*n* = 12)	Tenapanor 50 mg Chinese (*n* = 12)	Tenapanor 50 mg caucasian (*n* = 11)	Placebo (*n* = 12)	Total (*n* = 59)
Age (years)	26.7 ± 6.46	26.7 ± 4.60	28.6 ± 5.21	28.8 ± 4.51	26.2 ± 4.37	27.4 ± 5.04
Male (*n*, (%))	9 (75.0)	9 (75.0)	7 (58.3)	7 (63.6)	7 (58.3)	39 (66.1)
Height (cm)	163.92 ± 5.783	167.25 ± 10.376	162.96 ± 10.290	171.36 ± 9.440	166.92 ± 9.695	166.40 ± 9.415
Weight (kg)	59.42 ± 10.562	63.74 ± 7.754	57.80 ± 7.924	65.66 ± 10.196	62.57 ± 11.403	61.77 ± 9.761
BMI (kg/m^2^)	22.027 ± 3.1629	22.802 ± 2.2143	21.742 ± 1.9196	22.312 ± 2.4982	22.315 ± 2.3189	22.238 ± 2.3980
Subjects with any history of disease (*n*, (%))	1 (8.3)	1 (8.3)	1 (8.3)	1 (9.1)	2 (16.7)	6 (10.2)

*Note.* Data are shown as mean ± SD, unless otherwise specified.

**Table 2:** Main PK parameters of tenapanor-M1 following single and repeated dose administration of tenapanor in healthy subjects.

	10 mg, Chinese subjects (*n* = 12)		30 mg, Chinese subjects (*n* = 12)		50 mg, Chinese subjects (*n* = 12)		50 mg, Caucasian subjects (*n* = 11)	
	Geometric mean	Geo CV%	Geometric mean	Geo CV%	Geometric mean	Geo CV%	Geometric mean	Geo CV%
*Single dose*								
*T*_max_ (h)^a^	7.508 (4.00 to 10.00)		8.458 (2.00 to 9.95)		8.008 (3.97 to 10.05)		7.000 (4.00 to 10.00)	
*C*_max_ (ng/mL)	1.7294	36.84	3.4924	38.18	5.5194	36.30	5.2455	49.07
AUC_0-*t*_ (hour *∗* ng/mL)	34.1165	57.46	90.9420	52.73	169.6988	40.29	129.4200	63.81
AUC_0-∞_ (hour *∗* ng/mL)	63.4915^b^	40.56	116.0119	44.11	195.0912	36.61	152.4630	51.84
*t*_1/2_ (hour)	20.61^b^	30.40	18.82	26.47	18.54	24.65	16.37	22.36
CL/F (L/hour)	157.5015^b^	40.56	258.5942	44.11	256.2904	36.61	327.9484	51.84
*V*_*d*_/F (L)	4682.8820^b^	43.80	7021.5237	43.92	6855.9845	40.79	7745.8242	55.13

*Repeated dose*								
*T*_max,ss_ (h)^a^					1.975 (0.98 to 6.97)		4.000 (0.00 to 9.00)	
*C*_ss_ (ng/mL)					13.4617	29.89	12.1614	38.69
*C*_max,ss_ (ng/mL)					19.1772	26.31	17.6014	33.31
*C*_min,ss_ (ng/mL)					13.3380	30.11	12.1614	38.69
*C*_av,ss_ (ng/mL)					16.8094	26.05	15.2140	35.09
AUC_0-*τ*_ (hour *∗* ng/mL)					201.7129	26.05	182.5681	35.09
DF (%)					33.7463	23.97	34.8939	23.18
*t*_1/2_ (hour)					19.32	20.26	16.77	24.02
CL_ss_/F (L/hour)					247.8770	26.05	273.8704	35.09

AUC area under curve, CV coefficient of variation, DF degree of fluctuation, SD standard deviation. ^a^Median (range). ^b^*n* = 10.

**Table 3:** Linear analysis of pharmacokinetic parameters of different dose groups in Chinese healthy subjects.

*T*-test of linear regression coefficient							
PK parameters	Slope	Statistic (t)	*P*	90% CI	95% CI	Judgment interval^a^	Judgment interval^b^
*C*_max_	0.71	8.00	<0.0001	[0.56, 0.86]	[0.53, 0.89]	[0.86, 1.14]	[0.57, 1.43]
AUC_0-t_	0.98	8.36	<0.0001	[0.78, 1.18]	[0.74, 1.22]	[0.86, 1.14]	[0.57, 1.43]
AUC_0-∞_	0.68	6.59	<0.0001	[0.50, 0.85]	[0.47, 0.89]	[0.86, 1.14]	[0.57, 1.43]

*Note.* The linear pharmacokinetic characteristics of PK parameters were evaluated by power function model. The terminal elimination phase is linear, so AUC_0-∞_ with extrapolation area greater than 20% is also included in the model calculation. ^a^Adopt Smith standard: *θ*_*L*_ = 0.80 and *θ*_*H*_ = 1.25 [21]. ^b^Adopt Hummel standard: *θ*_*L*_ = 0.5 and *θ*_*H*_ = 2.0 [22]. Calculation of judgment interval: [1 + ln(*θ*_*L*_)/ln(*r*), 1 + ln(*θ*_*H*_)/ln(*r*)]. *r* = maximum dose/minimum dose.

**Table 4:** Summary of adverse events.

	Tenapanor 10 mg (*n* = 12)	Tenapanor 30 mg (*n* = 12)	Tenapanor 50 mg Chinese (*n* = 12)	Tenapanor 50 mg caucasian (*n* = 11)	Placebo (*n* = 12)
Participants experiencing at least 1 TEAE	5 (41.7%)	7 (58.3%)	12 (100%)	11 (100%)	6 (50.0%)
AEs occurring in at least 2 participants in any group					
Diarrhea	2 (16.7%)	0	12 (100%)	11 (100%)	1 (8.3%)
Increased systolic blood pressure	1 (8.3%)	3 (25.0%)	0	1 (9.1%)	0
Hypertriglyceridemia	1 (8.3%)	2 (16.7%)	0	0	1 (8.3%)
Hyperlipidemia	1 (8.3%)	0	2 (16.7%)	0	0
Fever	0	0	3 (25.0%)		1 (8.3%)
Abdominal distention	0	0	0	3 (27.3%)	0
Increased transaminases	1 (8.3%)	2 (16.7%)	0	0	0
Arrhythmia	0	2 (16.7%)	0	0	0

*AE* adverse event, *TEAE*treatment-emergent adverse event.

## Data Availability

The study is registered at https://www.chinadrugtrials.org.cn/ (no. CTR20201783). The data supporting the study findings are available from the corresponding author upon reasonable request.

## Supplementary Materials

Supplementary Materials Supplementary Figure 1: Dose and exposure relationship over the single-ascending dose range of 10 to 50 mg in healthy Chinese subjects. Filled circles and lines in orange indicate the theoretical exposure according to a proportional increase along with the dose levels, and those in blue indicate the actual exposure after single-ascending doses. Supplementary Figure 2: Dose and exposure relationship over the single- and multiple-ascending dose range of 15 to 180 (90) mg in Japanese healthy subjects from study D5611C00005. Filled circles and lines in orange indicate the theoretical exposure according to a proportional increase along with the dose levels, and those in blue indicate the actual exposure under single-ascending doses. Supplementary Table 1: Comparison of PK parameters of tenapanor-M1 in healthy Chinese and Caucasian subjects receiving 50 mg single and repeated dose of tenapanor.
