# Supplementary material for: Pharmacokinetics, Safety, and Tolerability of Tenapanor in Healthy Chinese and Caucasian Volunteers: A Randomized, Open-Label, Single-Center, Placebo-Controlled Phase 1 Study
Source: Int J Clin Pract. 2024 Mar 6;2024:1386980. doi: 10.1155/2024/1386980 (PMC10937073; doi:10.1155/2024/1386980)
Supplement: Supplementary Materials — Supplementary Figure 1: Dose and exposure relationship over the single-ascending dose range of 10 to 50 mg in healthy Chinese subjects. Filled circles and lines in orange indicate the theoretical exposure according to a proportional increase along with the dose levels, and those in blue indicate the actual exposure after single-ascending doses. Supplementary Figure 2: Dose and exposure relationship over the single- and multiple-ascending dose range of 15 to 180 (90) mg in Japanese healthy subjects from study D5611C00005. Filled circles and lines in orange indicate the theoretical exposure according to a proportional increase along with the dose levels, and those in blue indicate the actual exposure under single-ascending doses. Supplementary Table 1: Comparison of PK parameters of tenapanor-M1 in healthy Chinese and Caucasian subjects receiving 50 mg single and repeated dose of tenapanor. [file 1386980.f1.pdf]

Supplementary Figure 1 Dose and exposure relationship over the single-ascending dose range of 10 to 50 mg in healthy Chinese subjects. Filled circles and lines in orange indicate the theoretical exposure according to a proportional increase along with the dose levels, and those in blue indicate the actual exposure after single-ascending doses.

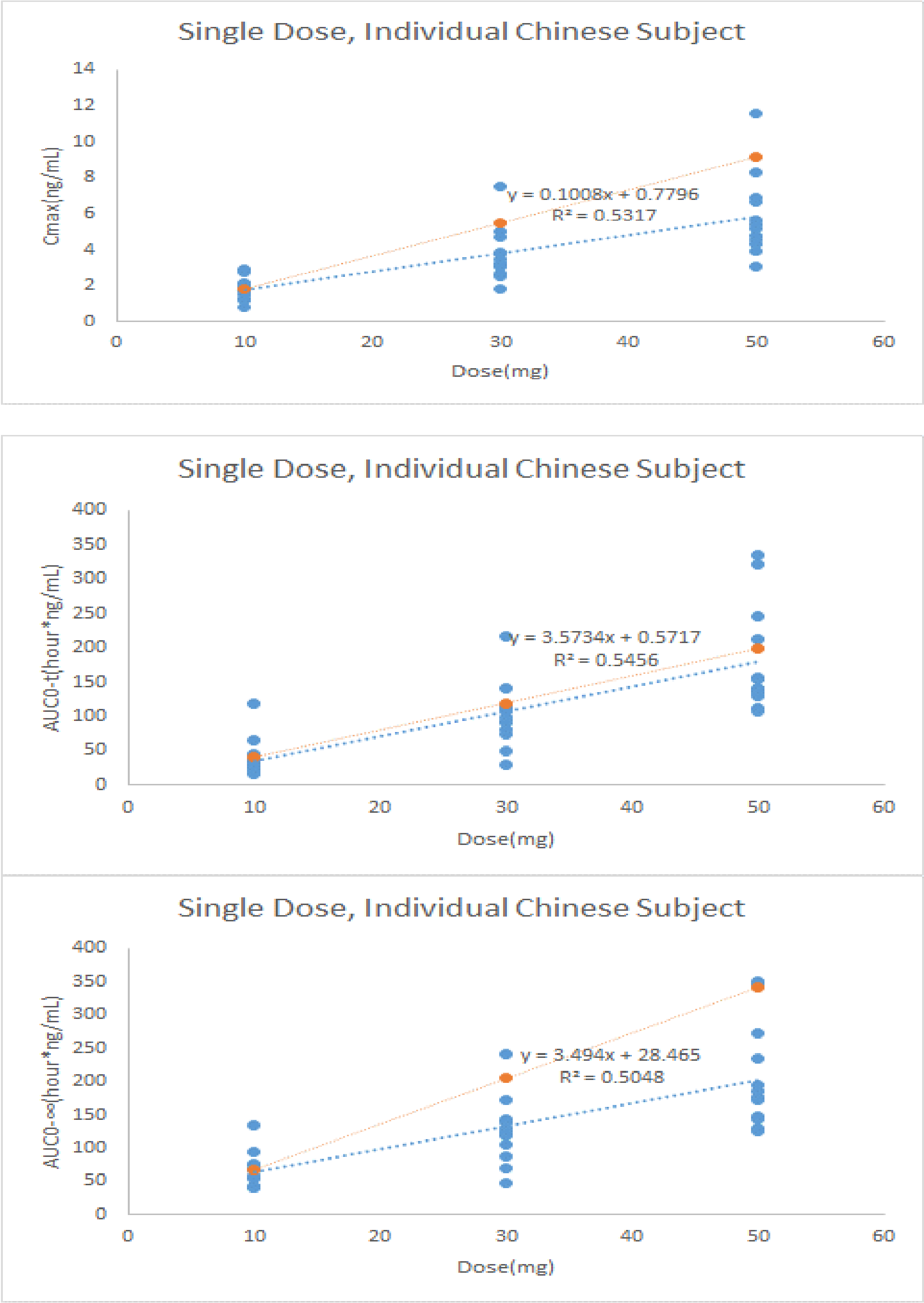

Supplementary Figure 2 Dose and exposure relationship over the single- and multiple-ascending dose range of 15 to 180 (90) mg in Japanese healthy subjects from study D5611C00005 (NCT02176252). Filled circles and lines in orange indicate the theoretical exposure according to a proportional increase along with the dose levels, and those in blue indicate the actual exposure under single-ascending doses.

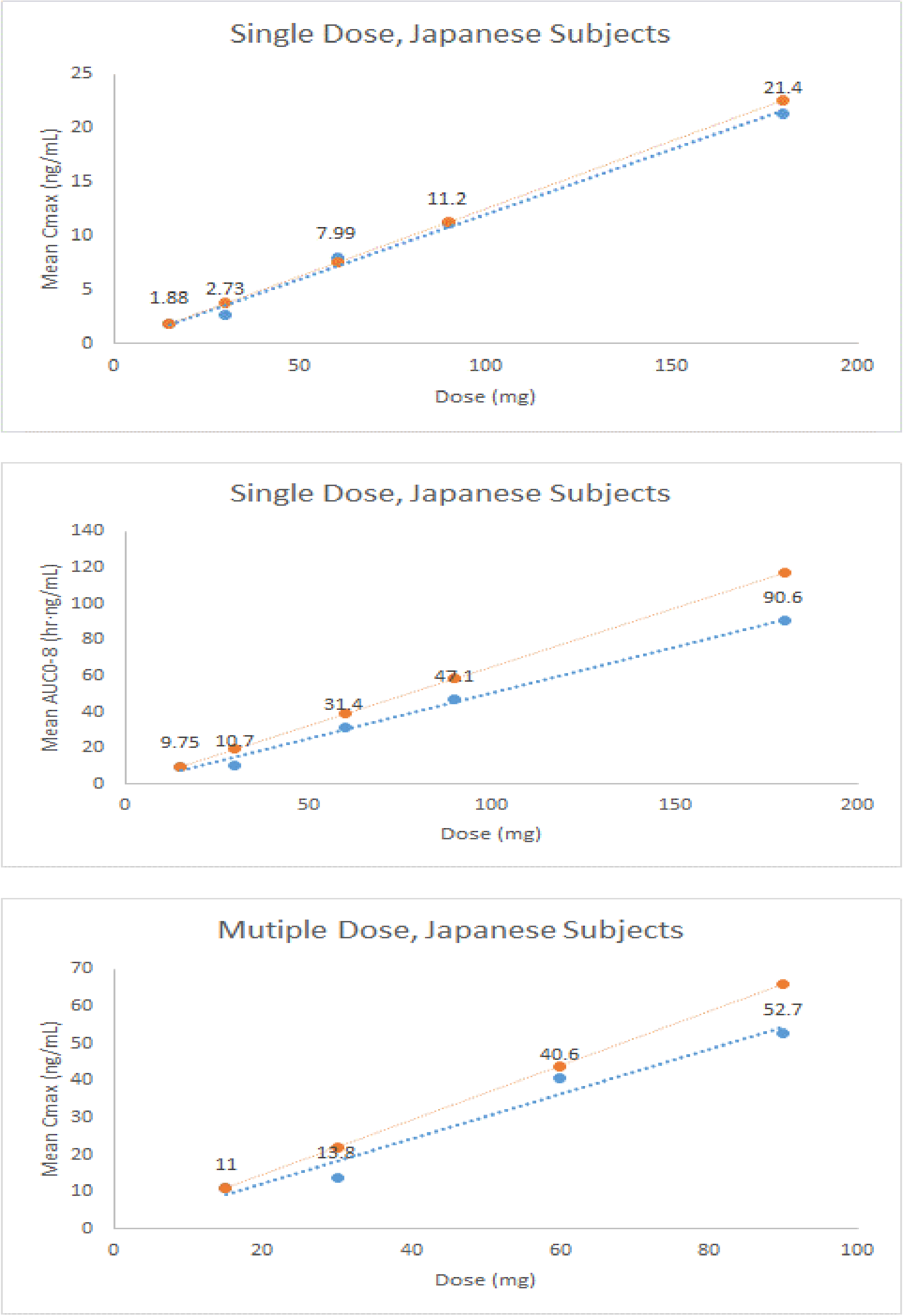

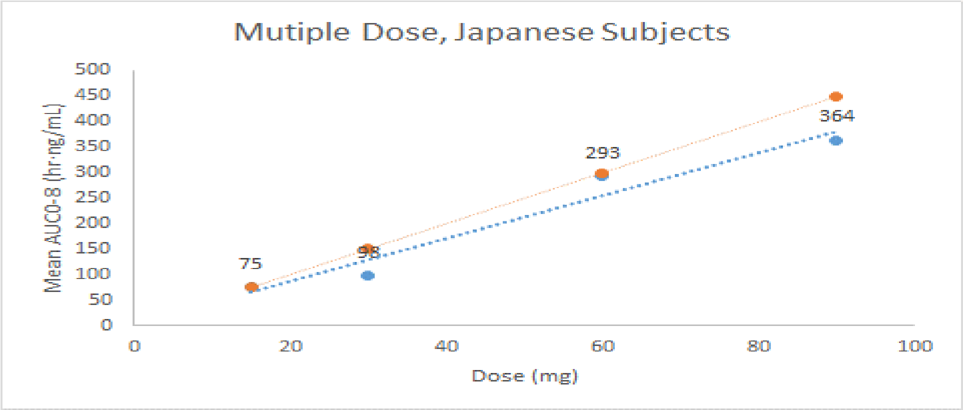

Supplementary Table 1 Comparison of PK parameters of tenapanor-M1 in healthy Chinese and Caucasian subjects receiving 50 mg single and repeated dose of tenapanor.

	Geometric mean and ratios			90% CI
	Chinese	Caucasian	Chinese/Caucasian	of Chinese/Caucasian
50 mg-Single dose				
C_{max} (ng/mL)	5.52 (n=12)	5.25 (n=11)	1.05	0.78-1.41
AUC_{0-t} (hour*ng/mL)	169.70 (n=12)	129.42 (n=11)	1.31	0.92-1.87
$AUC_{0-\infty}$ (hour*ng/mL)	195.09 (n=12)	172.30 (n=9)	1.13	0.84-1.53
50 mg-Repeated dose				
C_{ss} (ng/mL)	13.46 (n=12)	12.16 (n=11)	1.11	0.87-1.41
C_{max_ss} (ng/mL)	19.18 (n=12)	17.60 (n=11)	1.09	0.88-1.34
C_{min_ss} (ng/mL)	13.34 (n=12)	12.16 (n=11)	1.10	0.86-1.39
C_{av_ss} (ng/mL)	16.81 (n=12)	15.21 (n=11)	1.10	0.89-1.37
$AUC_{0-\tau}$ (hour*ng/mL)	201.71 (n=12)	182.57 (n=11)	1.10	0.89-1.37

AUC area under curve, *CI* confidence interval
